# LV outflow obstruction after repair of atrioventricular septal defect: an uncommon but challenging problem

**DOI:** 10.1093/icvts/ivab327

**Published:** 2021-12-15

**Authors:** Richard A Jonas

**Affiliations:** Cohen-Funger Professor of Surgery, George Washington University, Chief Emeritus Division of Cardiac Surgery, Children’s National Hospital, Washington, DC, USA

**Keywords:** Left ventricular outflow obstruction, Partial and complete atrioventricular septal defect, Recurrent outflow obstruction after surgical repair, Detachment of left atrioventricular valve

The study by Ivanov *et al.* [1] entitled ‘Incidence and management of the left ventricular outflow obstruction in patients with atrioventricular septal defects’ builds on the highly important paper by Fong *et al.* [2] published in 2020 that described the results of a large multi-institutional study of complete atrioventricular septal defect in Australia with follow-up over 25 years. In the latter paper, propensity-matched groups drawn from 819 patients who underwent surgery between 1990 and 2015 demonstrated that the risk of development of left ventricular (LV) outflow tract obstruction following repair of complete atrioventricular septal defect was not influenced by the surgical technique, whether double patch or modified single patch. The current report by Ivanov *et al.* is a single institutional study that focusses attention on the results of surgical reintervention for left ventricular outflow tract obstruction following previous repair of both partial and complete atrioventricular septal defect at Royal Children’s Hospital, Melbourne. The authors conclude that irrespective of surgical technique to relieve obstruction, there is a high incidence of subsequent recurrence of left ventricular outflow tract obstruction. And in keeping with many previous reports, the risk of developing left ventricular outflow tract obstruction is greater in patients with partial atrioventricular septal defect relative to complete atrioventricular septal defect. However, the risk of subsequent recurrence was similar with both complete and partial atrioventricular septal defect.

An interesting finding of the study is that the majority of patients presented with a simple subaortic fibromuscular membrane. More complex forms of obstruction such as accessory chords, valvular tags and diffuse and complex narrowing of the left ventricular outflow tract were much less common. Nevertheless, many patients underwent additional surgical procedures in addition to resection of a fibromuscular membrane including septal myectomy. For example, subaortic cords were resected in 37.5% of patients at the first left ventricular tract obstruction procedure. Seventeen percentage of patients at the first operation for left ventricular outflow tract obstruction underwent augmentation of the superior bridging leaflet while at the second left ventricular outflow tract operation 25% of patients had superior bridging leaflet augmentation. One patient developed severe left atrioventricular valve regurgitation and required reoperation when the reattached valve dehisced.

This is an excellent study for a number of reasons. A large number of patients, 730 in all were studied. Patients were accumulated over a very long time interval. For the 275 patients with partial atrioventricular septal defect the time interval was from 1975 to 2019 and for the 455 patients with complete atrioventricular septal defect between 1990 and 2019. Follow-up was 100% complete with a median follow-up time of 15 years for the partial group and 13 years for the complete atrioventricular septal defect group. Interestingly, there were no late deaths in the group with complete atrioventricular septal defect and only one in the partial group. During the long period of follow-up, only 24 patients required reoperation for left ventricular outflow tract obstruction. The median time between initial repair and the first left ventricular outflow tract reoperation was 2.6 years for the complete group and 4.4 years for the partial group. The interval to the second left ventricular outflow tract reoperation was longer with a median of 9 years.

In conclusion, the authors are to be congratulated on their review of a large cohort of patients followed over an extremely long time period. Left ventricular outflow tract obstruction is fortunately uncommon and appears to reflect the underlying substrate anatomy rather than being a consequence of surgical technique at the first procedure, as noted in the previous multi-institutional study that included these same patients. The study confirms the greater propensity of partial atrioventricular septal defect to develop left ventricular outflow obstruction relative to complete atrioventricular septal defect. Despite its rarity and usual presentation as a simple subaortic fibromuscular membrane, LV outflow obstruction is unfortunately difficult to eliminate and requires careful follow-up with an important risk of a second recurrence. 
